# The trials and tribulations of conducting an m-health pilot randomized controlled trial to improve oral cancer therapy adherence: recommendations for future multisite, non-drug clinical trials

**DOI:** 10.1186/s13104-019-4264-6

**Published:** 2019-04-15

**Authors:** Lahiru Russell, Michaela C. Pascoe, John F. Seymour, Sanchia Aranda, Phyllis Butow, Karla Gough, Penelope Schofield

**Affiliations:** 1 School of Nursing and Midwifery, Faculty of Health, Deakin University, Geelong, VIC Australia; 2 Institute of Sport, Exercise and Active Living, Victoria University, Footscray, VIC Australia; 3 Peter MacCallum Cancer Centre, Parkville, Melbourne, VIC Australia; 4 Royal Melbourne Hospital, Parkville, Melbourne, VIC Australia; 5 University of Melbourne, Melbourne, VIC Australia; 6 Cancer Council Australia, Sydney, Australia; 7 Department of Psychology, University of Sydney, Sydney, Australia; 8 Swinburne University of Technology, Hawthorn, VIC Australia

**Keywords:** m-health, Feasibility, Chronic myeloid leukemia, Supportive care trials

## Abstract

**Objective:**

Integrating mobile phone-based health (m-health) interventions into healthcare systems is one solution to improve access to services for the growing number of patients with chronic illness. Practical challenges such as poor recruitment and inadequate resource allocation can hamper the assessment of such interventions with clinical trial methodology. This paper highlights the challenges encountered during a pilot randomized controlled trial of an m-health medication adherence intervention and offers recommendations for future multi-site, non-drug clinical trials.

**Results:**

Eighteen patients were recruited to the study; eight were randomly allocated to the intervention arm. Intervention participants responded to their daily medication-reminder text messages, indicating that medication had been taken or not, and nurses were able to organize their calls around their workload. The trial closed prematurely primarily due to inadequate numbers of eligible patients; however, other potentially resolvable feasibility issues were identified. These included lack of infrastructure at study sites, poor screening data acquisition and management processes, and inexperience in conducting supportive care trials at participating sites. M-health intervention trials are designed to inform implementation of best supportive care practice. Adequate skills and infrastructure are research prerequisites that require careful consideration and sufficient investment for the successful execution of multi-site supportive care trials.

*Trial registration* Australian and New Zealand Clinical Trials Register: ACTRN12612000635864

## Introduction

Given the increasing number of people diagnosed with chronic disease [1, 2], the mounting pressure on health services and the fiscal restraints on health care [3], cost effective telehealth interventions integrated with targeted, direct clinical contact are urgently required [4]. Mobile phone-based health (m-health) interventions have shown promise in improving oral drug adherence [5], however most trials have been methodologically flawed and none have yet been conducted in populations with cancer [6]. Oral tyrosine kinase inhibitor therapy is the standard of care for patients with chronic phase myeloid leukemia (CML); however, continuous, daily dosing is required for an indefinite period, often lifelong, to ensure treatment efficacy [7, 8]. Thus, optimal medication adherence is critical for patients with CML, making this an important population in which to trial m-health interventions to improve medication adherence [5].

Therefore we developed the REMIND intervention package [9] to promote medication adherence and provide coaching in the self-management of side effects through mobile phone alerts, self-care advice, and nurse telephone consultations. REMIND consisted of two synergistically operating elements: nurse telephone consultations to promote medication adherence and provide coaching in adverse effect self-management; and a m-health system comprising individually-tailored mobile phone alerts, to which patients were to text responses indicating that medication had been taken or not, with tailored self-management advice based on self-reported, weekly symptom assessment and medication adherence.

To evaluate REMIND we commenced a pilot randomized-controlled trial. Eighteen patients were recruited before premature trial closure; the original target was 40 patients. This brief research note explores the feasibility issues encountered during the pilot testing of REMIND, as well as possible solutions.

## Main text

### Methods

This study was conducted across three cancer centres in Australia and received authorisation to begin from the respective Human Research Ethics Committees. A detailed description of participant selection criteria, outcome measures and data collection process are provided elsewhere [9]. Consenting patients were randomised (1:1) using a computer-generated randomisation chart.

The recruitment target was 40 participants over approximately four months. However, after 12 months, only 18 participants had been recruited which prompted study discontinuation due to process and resource issues. Table 1 provides a summary of the problems encountered and suggested solutions.

**Table 1:** Problems experienced during the pilot study of REMIND

	Problem	Suggested solution
Recruitment	Overestimation of the prevalence of patients diagnosed with CML within the past 2 years	Expand patient eligibility criteria to other conditions where medication adherence is critical for long-term survival; Add more recruitment sites
Infrastructure	Limited computer availability on site to train participants as per protocol	Use versatile and portable devices such as tablet computers for research purposes. Where necessary, allocate appropriate devices to each site for the duration of the study period
Staff expertise	Inadequate knowledge and understanding of supportive care research	Provide specific training in the purpose and conduct of supportive care studies for clinical trial staff; collaborate with other hospital departments more familiar with supportive care methodologies (e.g. psychology and psychiatry)
Site visit	Site initiation and monitoring conducted over the phone or by email	Adopt a combination of central and on-site visits; ideally, funding should support site initiation visits, then monitoring can be conducted centrally
Objective measurement of medication adherence	*Monitoring drug response* Difficulty in coordinating routine blood testing with study commencement and baseline assessment *Pill count* Unused meditation packs and prescription refills not returned Hospital was not the only place for meditation refills	Use of electronic pill monitoring device Conduct secondary database analysis
Data collection	Use of two separate online platforms for data collection	Use of a single software application to capture and manage clinical data (e.g. REDCap)

### Results and discussion

#### Recruitment

Despite the three participating sites initially confirming having sufficient CML patients to meet study timelines, most approached patients did not meet eligibility criteria. The overestimation of the number of patients meeting inclusion criteria, also known as Lasagna’s law [10, 11] is a very common phenomenon in clinical trials. To ensure adequate recruitment rates for medication adherence trials, patient inclusion criteria could be expanded to include other cancer types, other chronic disease types or clinical indications of unstable disease status. In addition, social networking could be employed to enable self-referral to trials targeting low-incidence diseases [12].

#### Site-related issues

Inadequate infrastructure at some participating sites, and a lack of understanding among staff about the focus of supportive care interventions affected study conduct and data collection. Specifically, the study protocol required that patients be trained in the REMIND program by a study nurse at the beginning of the intervention. However, limited computer availability in the clinic area prevented some patients from being trained as per protocol at one site, resulting in patient non-adherence to the intervention.

Another issue was the poor maintenance of clinic screening logs which are designed to record the recruitment of a consecutive sample of patients to inform on the representativeness of participants within the clinical setting [13]. Poor documentation of the screening and recruitment process significantly affected the quality of the information collected with only information from eligible participants’ being recorded. Without information about the total number of patients screened, ineligible, or declining participation, participants’ representativeness could not be established. Inadequate time and resource allocation may have prevented compliance with this process. Additionally, staff inexperience with supportive care studies may also have contributed to the problem. While they had extensive expertise in the management of clinical drug trials, some were unfamiliar with the purpose and priorities specific to supportive care trials, in particular aspects related to endpoints related to patient reported outcomes. From their experience with drug trial these outcomes were generally regarded as ‘an optional extra’. Specific training in the conduct of supportive care studies for clinical trial staff may be required and helpful to facilitate their conduct.

#### Site visits

Pre-study (or initiation) visits to recruiting centres are conducted to assess suitability of infrastructure and staff availability. Additionally, regular site monitoring visits are important to ensure that trial conduct complies with protocols and good clinical practice (GCP) guidelines [14]. The site-related issues outlined above may have been identified and addressed prior to trial initiation. However, budget restrictions only allowed for one site to receive an initiation visit, with no subsequent site monitoring visits. Therefore, the other two sites were initiated over the phone, and study monitoring was conducted over the phone and by emails. This method is not as effective as in-person visits, for which more time is allocated providing an opportunity to re-emphasise the importance of study processes, discuss study specificities, and resolve problems.

However, pre-study and regular site monitoring visits are costly [15], and adequate funding should be sought to conduct these visits. When necessary, study protocol and procedural compliance can be monitored through a combination of central and on-site monitoring [16]. Just as appropriate expertise and investment is required to implement best supportive care practice [17], the same should be required for the conduct of these trials. It is critical that relevant GCP content is conveyed through staff training, particularly emphasising aspects of the study design or protocol which may be unfamiliar to staff members who are inexperienced with supportive care trials. This could entail training data managers from Clinical Trials units or collaborating with other hospital departments, such as psychology, who may be more familiar with supportive care research methods.

#### Tracking medication adherence

In conjunction with self-reported medication adherence, we attempted to objectively assess adherence via BCR-ABL blood levels, a routinely performed test to monitor therapeutic drug response. However, the timing of BCR-ABL blood testing seldom coincided with baseline testing, which sometimes occurred 10 weeks prior to study commencement. Therefore, molecular testing was not a feasible endpoint. Other objective measures that were trialled in REMIND were pill counts and prescription refills. However, patients often forgot to return their used and unused medication, packs and prescription refill. Patients were also not consistently collecting their medication from the hospital, making tracking of prescription refills and pill counts an impossible task. Future studies should consider investing in medication adherence tracking devices such as electronic pill monitoring. Additionally, analyses of population-based health databases such as health insurances for medication claims could also provide an objective measure of medication adherence [18].

#### Data collection

Online data collection greatly reduces the costs of mailing out surveys, paying staff to remind patients to return completed surveys, and entering paper questionnaire data. However, even in this small study, four out of the 18 patients encountered problems using the online questionnaire when the support of a data manager was not available. Furthermore, the intervention patients had two sets of usernames and passwords; one set for the online data collection platform and another for the self-reported, weekly side-effects assessment, which caused confusion. The use of electronic surveys embedded on a single software application to capture and manage clinical data (e.g. REDCap [19]) would eliminate these problems for patients able to complete online surveys. Educating patients less familiar with online questionnaires through adequately trained staff members would keep costs to a minimum and reduce the risk of missing or incorrect data.

### Discussion

Clinical trials are crucial for the generation of evidence to effectively manage disease. Behavioural trials differ fundamentally from drug trials in the nature of their interventions, but the methodologies to establish efficacy and effectiveness are the same [20]. Interventions in behavioural trials are usually more complex to define and standardise than those of drug trials. A main reason is the variation in participants’ characteristics and preferences, which will influence their behaviour (e.g. medication adherence), and have an important impact on the estimated effect of the intervention [21]. Institutions and organisations conducting clinical trials usually have staff experienced in managing industry sponsored trials. Clinical trial staff are regularly trained in the good conduct of these studies, and are frequently monitored for adequate data collection and reporting. Considerable time and money are invested to ensure robust infrastructure and rigorous trial conduct [22, 23]. The same rigour is difficult to maintain in publicly funded studies with leaner budgets [20, 24]. Failing to provide adequate training may lead to under-reporting, and possibly study discontinuation [10, 25].

Despite the great potential that technology presents in delivering timely and cost-efficient supportive care programs for people requiring ongoing treatment, adequate resources to evaluate the efficacy of these intervention is imperative. If clinical trials units are to be allocated funding to conduct supportive care studies, careful evaluation of staff experience with, and infrastructure available to accommodate, supportive care studies is essential. It is important to continue to consider how to improve support for non-drug clinical trial research.

## Limitations

A limitation of this study is the small sample of nurses and patients used to pilot test this intervention. As CML is a relatively rare disease, it was difficult and time consuming to recruit patients. Future trials should consider assessing the impact of telehealth packages upon oral medication adherence in different cancer types.

## Abbreviations

CML: Chronic myeloid leukemia
GCP: Good clinical practice
M-health intervention: Mobile phone-based health intervention
